# Non-linearities in the Rod and Cone Photoreceptor Inputs to the Afferent Pupil Light Response

**DOI:** 10.3389/fneur.2018.01140

**Published:** 2018-12-21

**Authors:** Pablo Alejandro Barrionuevo, J. Jason McAnany, Andrew J. Zele, Dingcai Cao

**Affiliations:** ^1^Instituto de Investigación en Luz, Ambiente y Visión, Consejo Nacional de Investigaciones Científicas y Técnicas–Universidad Nacional de Tucumán, San Miguel de Tucumán, Argentina; ^2^Department of Ophthalmology and Visual Sciences, University of Illinois at Chicago, Chicago, IL, United States; ^3^Visual Science Laboratory, School of Optometry and Vision Science & Institute of Health and Biomedical Innovation, Queensland University of Technology, Brisbane, QLD, Australia

**Keywords:** retina, ERG analysis, pupil, photoreceptors cells, beats, mesopic light level, non-linearity

## Abstract

**Purpose:** To assess the nature and extent of non-linear processes in pupil responses using rod- and cone-isolating visual beat stimuli.

**Methods:** A four-primary photostimulating method based on the principle of silent substitution was implemented to generate rod or cone isolating and combined sinusoidal stimuli at a single component frequency (1, 4, 5, 8, or 9 Hz) or a 1 Hz beat frequency (frequency pairs: 4 + 5, 8 + 9 Hz). The component frequencies were chosen to minimize the melanopsin photoresponse of intrinsically photosensitive retinal ganglion cells (ipRGCs) such that the pupil response was primarily driven by outer retinal photoreceptor inputs. Full-field (Ganzfeld) pupil responses and electroretinograms (ERGs) were recorded to the same stimuli at two mesopic light levels (−0.9 and 0 log cd/m^2^). Fourier analysis was used to derive the amplitudes and phases of the pupil and ERG responses.

**Results:** For the beat frequency condition, when modulation was restricted to the same photoreceptor type at the higher mesopic level (0 log cd/m^2^), there was a pronounced pupil response to the 1 Hz beat frequency with the 4 + 5 Hz frequency pair and rare beat responses for the 8 + 9 Hz frequency pair. At the lower mesopic level there were few and inconsistent beat responses. When one component modulated the rod excitation and the other component modulated the cone excitation, responses to the beat frequency were rare and lower than the 1 Hz component frequency condition responses. These results were confirmed by ERG recordings.

**Conclusions:** There is non-linearity in both the pupil response and electroretinogram to rod and cone inputs at mesopic light levels. The presence of a beat response for modulation components restricted to a single photoreceptor type, but not for components with cross-photoreceptor types, indicates that the location of a non-linear process in the pupil pathway occurs at a retinal site earlier than where the rod and cone signals are combined, that is, at the photoreceptor level.

## Introduction

The response of the pupil to radiance information, the “pupil light reflex” (PLR), is mediated by phototransduction in rods, cones and by the photopigment melanopsin that is expressed in intrinsically photosensitive retinal ganglion cells (ipRGCs) (1–4). The olivary pretectal nucleus (OPN) commands pupillary movements, and it receives afferent signals from ipRGCs (2, 5, 6). Classical PLR studies used two types of stimulation paradigms, including pulsed and flickering stimulation. The best known is the PLR to a pulse of light, in which, roughly, two main stages in the temporal domain can be identified: the transient (or phasic) stage and the tonic (or sustained) stage. This pulsed PLR paradigm has revealed that cones are prevalent in the phasic stage, while rods and melanopsin are mostly conducting the tonic response (7–9). Another approach includes analyzing pupillary responses to flickering stimulation in the frequency domain through Fourier transformation (10–12). With this approach, it is determined that melanopsin, rods, L- and M-cones provide excitatory input to the pupil pathway, whereas S-cones provide inhibitory inputs (10, 12, 13). This is consistent with the spectral characteristics of primate ipRGCs receptive fields (14), although recent studies reported inhibitory responses for M-cones inputs (15, 16). Cone contributions to the flicker pupil response summate linearly with rod and/or melanopsin contributions (11), and melanopsin is combined linearly with luminance information (L + M + S) and [(L + M) – S] chromatic signals (10). However, a non-linear “winner takes all” mechanism has been identified with predominant participation of rods and melanopsin (8, 17), and this type of mechanism seems to account for the combination of melanopsin and (L – M) chromatic signals (10). Besides this evidence, the non-linear properties of rod and cone inputs to the pupil response were rarely investigated.

A tool to study non-linear mechanisms in the afferent pupillary pathway is through beat responses, which are a signature of non-linear processing (18). When two sinusoidal stimuli of different frequencies are processed by a non-linear system a response appears with a frequency corresponding to the difference of those frequencies; this phenomenon is called a beat. Oscillations at the beat frequency therefore reveal that the system is responding non-linearly to the stimulation. Beat responses have been used to study non-linearities in the auditory system (19) and in vision for example, to study binocular interactions (20, 21).

Non-linearities in the pupil pathway have been suggested in the retina or iris muscle (22, 23). Howarth and colleagues (22) used a beat paradigm with monocular and dichoptic stimulation and inferred that the site of the non-linearity preceded the locus where signals from the two eyes are integrated. Retinal non-linearities can account for the effects of saturation and rectification in cell responses (24). Saturation is caused by the limited dynamic range of retinal cells whereas rectification causes a cell response to sinusoidal stimulation (positive or negative) to be excluded or inverted (25, 26). The presence of beats in electroretinogram (ERG) recordings, has been attributed to rectification within the outer retina (18).

The purpose of this study was to isolate non-linear processes in the afferent pupil responses to rod and cone inputs using visual beat stimuli. If a beat response is observed in both the pupil light response and electroretinogram, the origin of the non-linearities will likely be in the retina.

## Methods

### Observers

Three male observers (age 24–43 years) participated in the study. All have normal color vision (assessed by the Neitz OT anomaloscope and the Farnsworth-Munsell 100-hue test). Ophthalmological examinations excluded any retinal or optic nerve condition that could affect the results. The study protocols were approved by the Institutional Review Board at University of Illinois at Chicago and adhered to the tenets of the Declaration of Helsinki.

### Apparatus

A ColorDome Ganzfeld in an Espion^3^ electrophysiology system (Diagnosys LLC, Lowell, MA, USA) was used for stimulus presentation. We used the “dim ring” of LEDs in the ColorDome Ganzfeld to produce light levels within mesopic range. The “dim ring” had 4 LEDs with dominant wavelengths as 470 nm (“blue”), 524 nm (“green”), 588 nm (“amber”), and 636 nm (“red”) nm. The ColorDome Ganzfeld was programmed to serve as a four-primary photostimulator that could control the excitations of rods and three types of cones (S-, M-, and L-cones) independently using silent substitution (27). The cone excitations were computed based on the Smith-Pokorny cone fundamentals for the CIE 1964 10° Standard Observer (26). The cone chromaticities were described in a relative cone-troland space, which plots S/(L + M) vs. L/(L + M) (28). For an equal-energy-spectrum (*EES*) light, the L/(L + M) value is 0.667 and the S/(L + M) value is 1.0. The cone luminance is the sum of the L and M cone excitations and is specified in photopic cd/m^2^. Rod excitation was computed based on the scotopic luminous efficiency function, V′(λ), with normalization such that 1 photopic cd/m^2^ of *EES* light defines rod excitation of 1 rod cd/m^2^.

Since the built-in calibration provided by the Diagnosys system was based on the CIE 1931 2° standard observer, we calibrated the light outputs from the ColorDome LEDs so that we could specify stimuli in the CIE 1964 10° colorimetric system. The spectral distribution of each LED was measured with a PhotoResearch PR-670 spectroradiometer. The CIE 10° luminance of each LED at its maximum were calculated from the spectral measurements.

Pupil responses were recorded by an EyeLink II eyetracker (SR Research) at a 250 Hz sampling rate. The Espion^3^ electrophysiology system controlling the ColorDome triggered the eyetracker to synchronize the stimulation presentation and recording. Full-field electroretinograms (ERGs) were recorded in the Espion^3^ electrophysiology system with bandwidths of 0.3 and 300 Hz at a 2,000 Hz sampling rate using *DTL Plus* corneal electrodes, which were referred to ear clip electrodes and a wrist electrode ground. Head position was maintained using a chin rest in front of the ColorDome stimulator.

### Stimuli

**Figure 1:**
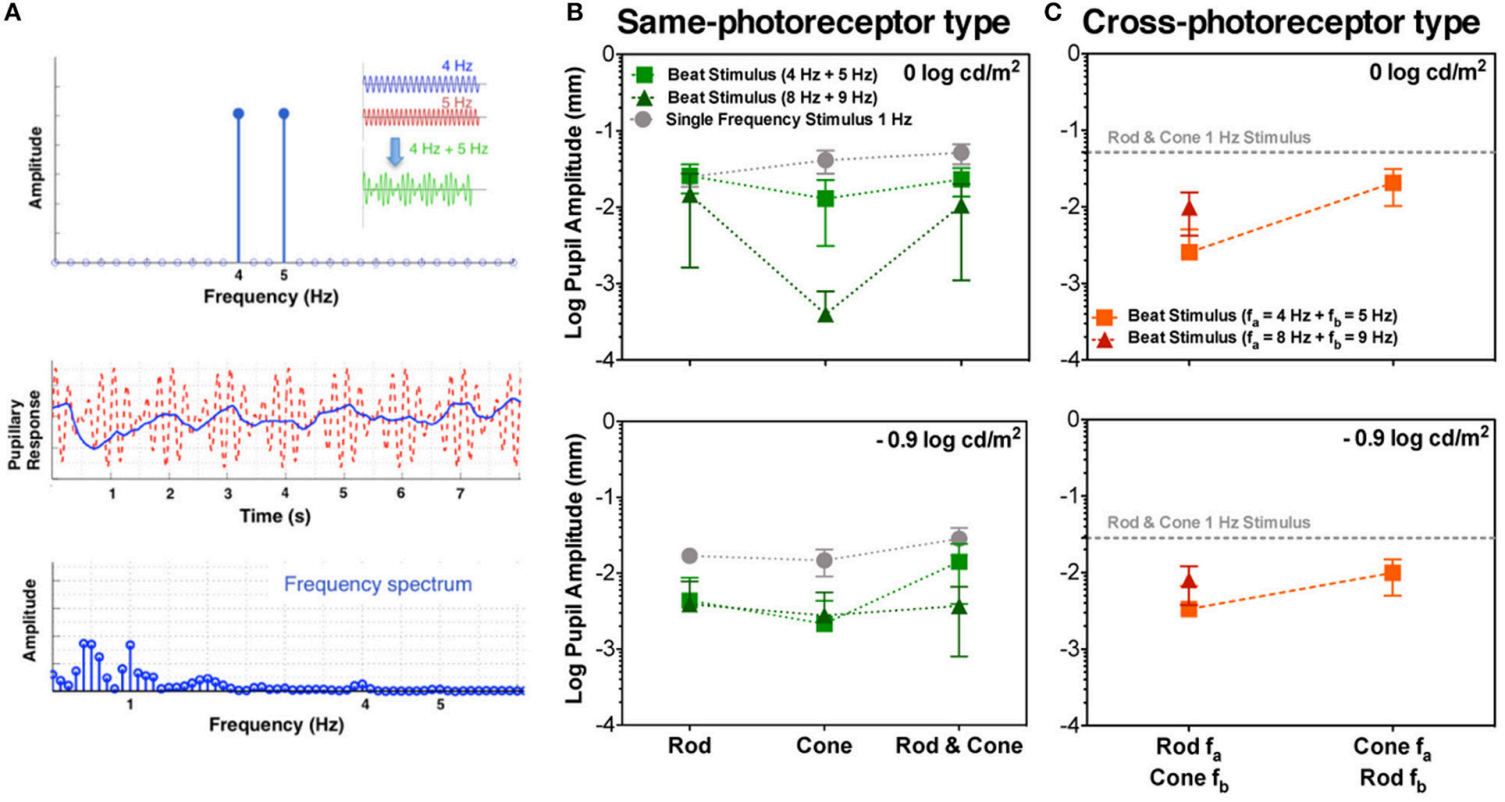
Beats in the pupillary responses. **(A)** Linear system frequency response for combined sinusoidal stimulations at 4 and 5 Hz (top panel; temporal profile in the inset). Middle and bottom panels contain responses of S1 to Rod & Cone 4 Hz + Rod & Cone 5 Hz in the temporal and frequency domains, respectively. Averaged beat responses across participants are shown for combined 4 and 5 Hz (squares), and combined 8 and 9 Hz (triangles) in comparison with pupil responses for 1 Hz stimulation (circles) for same-photoreceptor type condition **(B)**, and Cross-photoreceptor type condition **(C)**. Error bars represent SEM.

We generated three types of photoreceptor-isolated sinusoidal stimuli at two mesopic light levels: (1) isolated rod stimuli (“Rod,” only rod excitation was modulated while maintaining constant cone excitations), (2) isolated cone luminance stimuli (“Cone,” only cone luminance, L + M, was modulated while maintaining constant rod excitation), and (3) combined rod and cone stimuli (“Rod & Cone,” both rod and cone luminance signals were modulated in phase). To achieve a large contrast range for both the rod or cone modulations, the time-averaged chromaticity was set to L/(L + M) = 0.77 and S/(L + M) = 0.20 in a relative cone troland space (27). The time-averaged photopic luminances were −0.9 log cd/m^2^ (0.13 photopic cd/m^2^ or 0.10 scotopic cd/m^2^ or 11 log quanta/cm^2^/s) or 0 log cd/m^2^ (1.0 photopic cd/m^2^ or 0.82 scotopic cd/m^2^ or 11.9 log quanta/cm^2^/s), in order to minimize the melanopsin contribution. The low adaptation luminance was achieved by covering the ColorDome with a calibrated 0.9 log unit neutral density filter. The rod and/or cone excitations were sinusoidally modulated at 25% Michelson contrast. For pupil measurements, the stimuli were modulated at one frequency at 1, 4, 5, 8, or 9 Hz alone (i.e., component frequency condition), or at two frequencies with the same phase (i.e., beat frequency condition). The frequency pairs (4 + 5 Hz, or 8 + 9 Hz) generated a 1 Hz beat frequency, the optimal beat frequency for the pupil light response (22). A beat stimulus in the temporal domain is shown in Figure 1A (top panel). The component frequencies were chosen because at these frequencies, melanopsin sensitivity is minimal (29). Although the pupil response was weak, the photoreceptor response was still measureable (11). The beat stimuli could be the combination of the same photoreceptor types or different photoreceptor types (Table 1).

**Table 1:** Beat conditions tested.

	**Rod 4 Hz**	**Cone 4 Hz**	**Rod and Cone 4 Hz**
**Rod 5 Hz**	Same-	Cross-	
**Cone 5 Hz**	Cross-	Same-	
**Rod and Cone 5 Hz**			Same-
	**Rod 8 Hz**	**Cone 8 Hz**	**Rod & Cone 8 Hz**
**Rod 9 Hz**	Same-	Cross-	
**Cone 9 Hz**	Cross-	Same-	
**Rod and Cone 9 Hz**			Same-

*Photoreceptor type combinations assessed for 4 + 5 Hz pairs (top) and 8 + 9 Hz pairs (bottom)*.

### Procedure: Pupil Response and ERG Recording

The pupil response and ERGs were recorded binocularly in separate sessions. Each pupil recording session started with 30 min of dark adaptation and included two mesopic light levels (0.9 log cd/m^2^ followed by 0 log cd/m^2^). For each light level, the observers adapted to a steady background for 2 min before recording. Data were collected over 10 s period for a trial with a 10 s interval between trials. Each session lasted ~1.5 h. Sufficient rest was given between conditions.

For the binocular ERG recording, both eyes were dilated with 1% tropicamide drops and dark-adapted for 15 min before ERG measurements. The same photoreceptor isolating stimuli used with the pupil recordings were used for the ERG measurements with the combination of 4 and 5 Hz frequencies. The recording procedure was similar to the pupil recordings. Individual trials that included an eye movement or blink artifact (i.e., maximum amplitude ≥ 200 μV) were removed automatically by the Diagnosys Espion^3^ electrophysiology system or manually by the ERG technician during the recordings. Fifteen sweeps were recorded for each condition. One session lasted ~1.5 h. Each session was repeated three times on different days.

### Data Analysis

For all stimulus conditions, the pupil or ERG responses from the two eyes of each observer were similar and the data from the two eyes were averaged. The averaged waveform for each condition at a light level was subjected to a discrete Fourier transformation (2,500 samples) to extract the amplitude and phase of the first harmonic. Noise for the pupil responses was estimated in the frequency domain from the component frequency conditions: 4, 5, 8, and 9 Hz, averaging the component amplitude obtained at 1 Hz in each case. For the ERG experiment, noise was estimated based on the amplitudes of a test frequency with a steady background light. The difference in the extracted amplitude and noise amplitude for each condition was computed for each observer. If the amplitude was smaller than the noise level for a condition, the amplitude for that condition was set as zero for further statistical analysis. The data were summarized as mean and standard error (SEM). Then the amplitudes with noise removed were compared using repeated measures ANOVA or paired *T*-test.

## Results

### Pupillary Recordings

**Figure 2:**
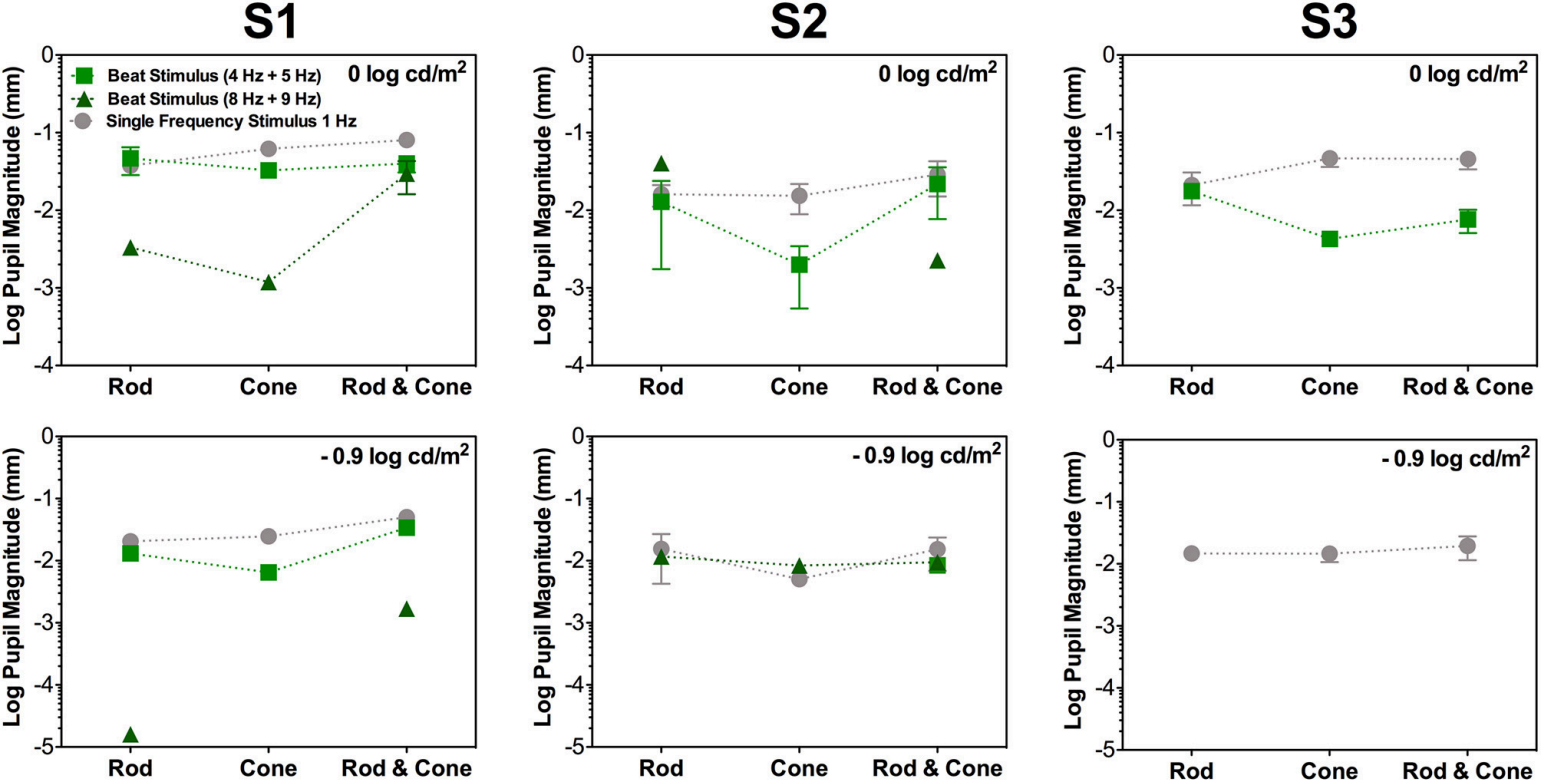
Data for the same photoreceptor type condition for individual participants; S1 (left column), S2 (middle column), and S3 (right column). Upper panels contains data for 0 log cd/m^2^, lower panels for −0.9 log cd/m^2^. Error bars represents SEM. For this condition thee three participants elicited beat responses. S1's beat responses were elicited in all cases except for Cone 8 Hz + Cone 9 Hz at −0.9 log cd/m^2^. For S2 (middle column), beat responses were elicited in all conditions for 4 + 5 Hz pair at 0 log cd/m^2^ and only for Rod & Cone condition at −0.9 log cd/m^2^. For 8 + 9 Hz pair no beat responses were obtained for Cone condition at 0 log cd/m^2^. For S3 (right column), no beat responses were elicited at −0.9 log cd/m^2^ or for 8 + 9 Hz stimuli at 0 log cd/m^2^.

A typical pupil response of one participant obtained for the beat frequency condition with the combined Rod & Cone 4 Hz + Rod & Cone 5 Hz at 0 log cd/m^2^ is shown in Figure 1A (middle panel for the temporal domain and bottom panel for the frequency domain). A pupillary response at the 1 Hz beat frequency is apparent when the stimulation is a combination of signals at 4 and 5 Hz; a linear system cannot produce a response at this beat frequency, indicating a non-linear process in the afferent pupil response. Average pupil amplitude responses of the 1 Hz component for the three participants at two mesopic light levels are shown in Figures 1B,C. Beat pupil responses were evident with the 4 + 5 Hz stimulus pairs for all photoreceptor types (Rod, Cone, or Rod & Cone) in the three participants at 0 log cd/m^2^, and only for S1 at −0.9 log cd/m^2^ (square symbols, Figure 2). Rod-Cone phase differences were similar for the beat frequency condition (15.74 ± 7.03°) and component frequency condition [10.94 ± 3.66°, *t*_(4)_ = 0.605, *p* = 0.58].

Considering the different photoreceptor combinations at 8 and 9 Hz at 0 log cd/m^2^ (Figure 1B, upper panel), the averaged beat responses were present for participants S1 and S2 only (Figure 2). At lower light level, the amplitude of the responses were reduced and the differences between beat responses and single frequency responses were not significant for the three photoreceptor combinations, *F*_(2,12)_ = 2.95, *p* = 0.128 (Figure 1B, lower panel). Individual results showed very small or null responses with the three combinations for participant S1 and null responses for S3 (Figure 2).

**Figure 3:**
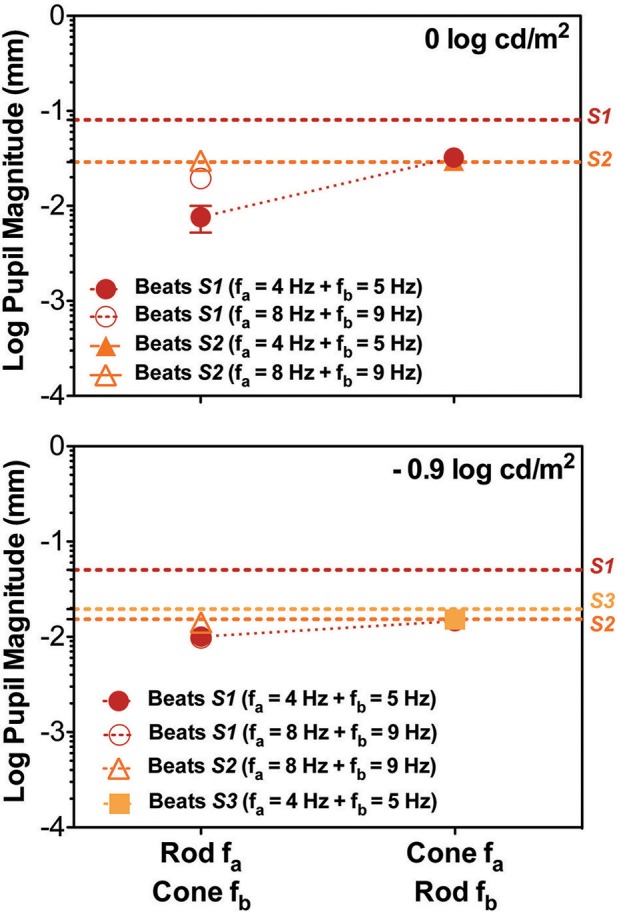
Data for the cross photoreceptor type condition for individual participants; S1 (red circles), S2 (orange triangles), and S3 (light orange squares). Dashed lines represents the Rod & Cone 1 Hz single frequency data for each subject. Upper panel contains data for 0 log cd/m^2^, lower panel for −0.9 log cd/m^2^. Error bars represents SEM. Cross-photoreceptor type results were rare and reduced with respect to Rod & Cone 1 Hz data. S1's pupillary recordings showed cross beat responses for both light levels. For S2 beat responses were obtained for Rod 8 Hz combined with Cone 9 Hz at both light levels, and for combination of Cone 4 Hz and Rod 5 Hz at 0 log cd/m^2^. For S3, beat responses were obtained when Cone 4 Hz was combined with Rod 5 Hz at the lower light level. Beat amplitudes for S2 and S3 were comparable to results for Rod & Cone 1 Hz stimulus.

Finally, beat stimuli modulating the cross photoreceptor types elicited beat responses in few cases (Figures 1C, 3). Participant S1's pupillary recordings showed beat responses for both light levels, however, for S2, beat responses were obtained for the Rod 8 Hz combined with Cone 9 Hz at both light levels, and for combination of Cone 4 Hz and Rod 5 Hz at 0 log cd/m^2^, whereas, S3's beat responses were obtained when Cone 4 Hz was combined with Rod 5 Hz at the lower light level (Figure 3). No response were obtained for Cone 8 Hz + Rod 9 Hz at both light levels for any participant (Figures 1C, 3).

**Figure 4:**
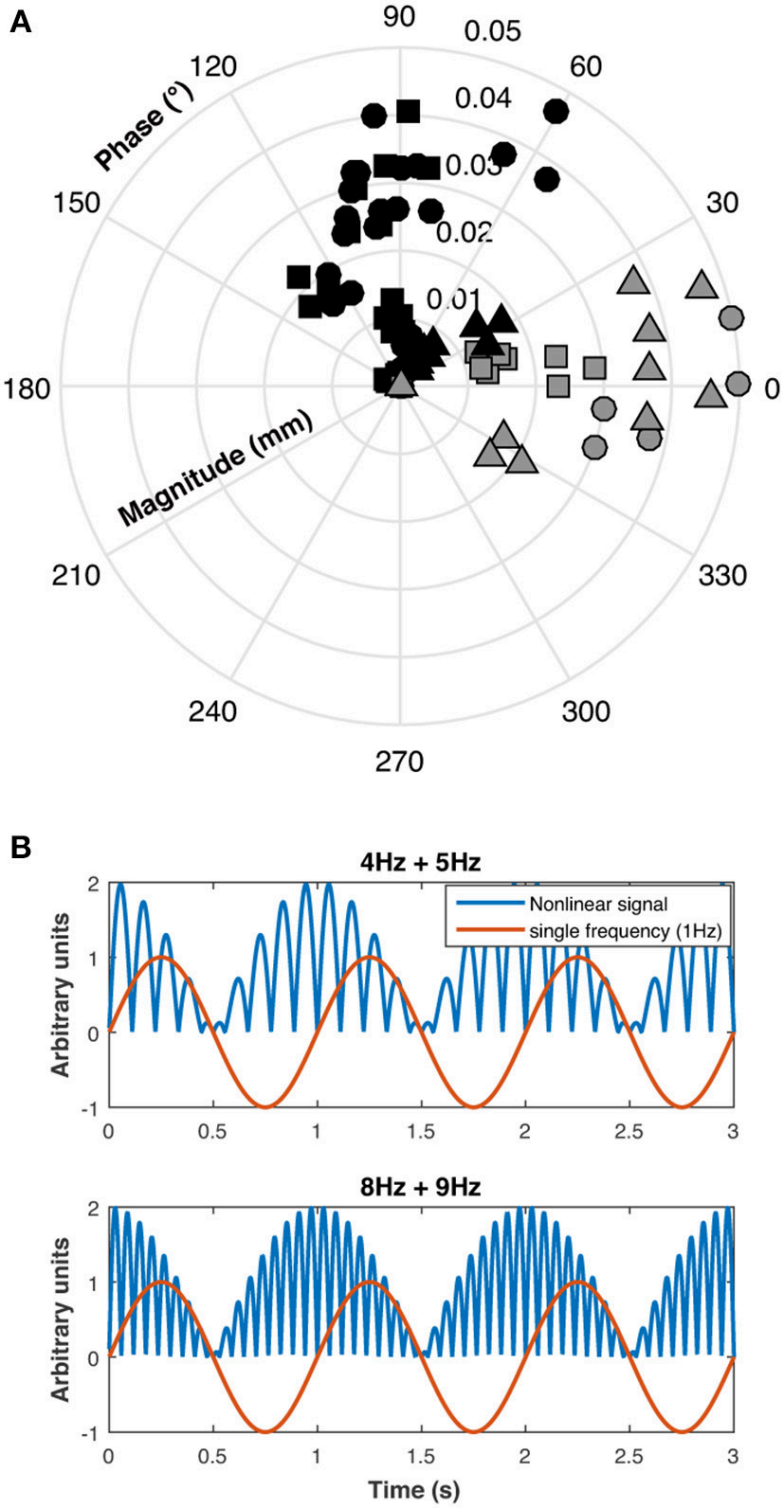
**(A)** Polar plot of individual results for the three participants (S1: circles, S2: squares, and S3: triangles) for beat responses (black symbols) and single 1 Hz responses (gray symbols), **(B)** This panel shows that beat phase caused by rectification is shifted 90° with respect to single frequency phase.

A polar plot of the pupillary responses of the three participants are shown in Figure 4A. The phase of the beat responses are shifted 90° with respect to the single frequency response phases, which is evidence for a non-linearity in the pupillary signal processing, possibly due to rectification which introduces a similar phase shift between the beat and component frequencies (Figure 4B).

### ERG Recordings

To determine if beat responses observed in pupillary measurements occurred at the retinal level, a second experiment was conducted by obtaining the ERG recordings of the three participants using similar stimuli modulations (4 Hz + 5 Hz) for the same-photoreceptor types.

**Figure 5:**
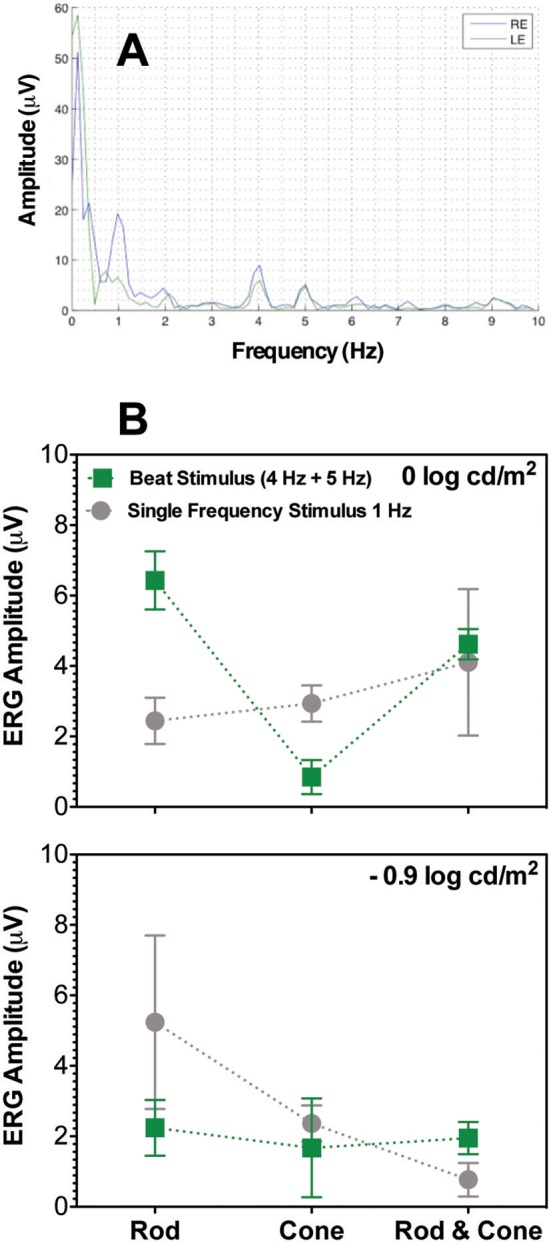
Beats in ERG recordings. **(A)** Frequency beat response of S1 to Rod & Cone 4 Hz + Rod & Cone 5 Hz condition. **(B)** Averaged beat responses for combined 4 and 5 Hz (squares) in comparison with results for single 1 Hz stimulation. Error bars represent SEM.

The frequency profile of the ERG amplitudes for participant S1 at 0 log cd/m^2^ for the combined Rod & Cone 4 Hz + Rod & Cone 5 Hz condition (Figure 5A) shows clear peaks appear at 1, 4, and 5 Hz. Beat ERG responses were obtained for most cases. Figure 5B shows the averaged results for the three participants. At both 0 log cd/m^2^ (Figure 5B, upper panel) and −0.9 log cd/m^2^ (Figure 5B, lower panel), the beat responses were similar to the single frequency responses [*F*_(1,8)_ = 0.85, *p* = 0.4; *F*_(1,8)_ = 0.5, *p* = 0.52; respectively]. The pattern of the ERG data was generally consistent with the pupillary responses.

## Discussion

Substantial pupillary beat responses were obtained for combined sinusoidal stimulations at 4 Hz and 5 Hz of the same-photoreceptor type (Rod 4 Hz + Rod 5 Hz, Cone 4 Hz + Cone 5 Hz, and Rod & Cone 4 Hz + Rod & Cone 5 Hz) at the higher mesopic light level (0 log cd/m^2^), and were consistent across participants. Beat responses observed in the 4 + 5 Hz pupil response were similarly obtained in ERG recordings, confirming that non-linearities are present at the retinal level. According the analysis of the phase difference between beat data and single 1 Hz data (Figure 4), a rectification process may be involved. At the lower mesopic light level, the beat responses were inconsistent, likely because at this illumination level the signal-to-noise ratios are decreased and the cones are at the lower end of their operating range. This was also evident for the same-photoreceptor combination type with the 8 and 9 Hz stimulation (at both light levels). A similar outcome was observed for the cross-photoreceptor type condition. However, for participant S1 beat responses were obtained in most cases, meaning that individual differences are important in retinal non-linearities to elicit beat responses. Individual differences as those found in our study can emerge from many sources, such as fatigue, emotional states, other sensory inputs and refractive errors (30, 31). It will be interesting to investigate this issue in the future.

A previous study evaluating pupillary beat responses used higher frequency stimuli in the range of 10–25 Hz (22). These authors claimed this frequency range is optimal for binocular conditions, however we found strong beats in the 4–5 Hz range and weaker beat responses in the range of 8–9 Hz. It is known that the flicker pupil light response has a cut-off resolution frequency in the order of ~8–9 Hz (11, 32–34), but other factors, such as the conditions in which the experiments were carried out could explain this difference. They used a brighter photopic background (~15,000 photopic td) compared to our dimmer mesopic lighting (~10 photopic td), had a higher modulation depth (~80 vs. 25% for this study), and used broadband lights. As such, we observed beat responses for same-photoreceptor type condition, and the amplitude of the beat response could be larger with higher light levels for cones activation. We did not run experiments for source frequencies higher than 9 Hz, so we cannot rule out the appearance of beats at higher frequencies as those used by Howarth and colleagues (22). More research is needed to understand the relationship between light level and the optimal frequency range to modulate non-linear responses.

Since the discovery of intrinsically photosensitive retinal ganglion cells (ipRGCs) in mammals (5, 14, 35), understanding of the retinal circuit to control pupillary response to light has been advanced. From the five types of ipRGCs detected in the rodent retina (36, 37), M1 cells disproportionally innervate the OPN (5, 38). In primate retinas, outer cells are the counterparts of the rodent M1 cells (39). Outer cells have their dendrites in the OFF sublamina of the interplexiform layer near the inner nuclear layer and are innervated by bipolar and amacrine cells (40, 41). It was suggested that diffuse bipolars DB6 convey excitatory inputs to L and M cones, while dopaminergic amacrines convey major inhibitory signals (10, 40, 42). Considering the pathways conveying rods signals, it was shown that there is no direct innervation of rod bipolar cell to ipRGCs (39). In primates rod and cone signals are combined at the outer retina through the rod-cone gap junction pathway, at the inner retina through the rod->rod bipolar->AII amacrine->cone bipolar pathway, and potentially through horizontal cells feedback between photoreceptors (43, 44). For pupillary responses it was suggested that the most probable pathway to activate phasic pupillary movements is via rod-cone gap junctions->DB6 bipolar cells (10).

Our results showed weaker and more sparse beats in the cross-photoreceptor type condition than in the same-photoreceptor type condition. Therefore, same-photoreceptor non-linear processing produced stronger signals (able to evoke pupillary movements) than cross-photoreceptor non-linear processing, which in turn means that the non-linearities occur before rod and cone signals interact. Since beat responses were also obtained in the ERG measurements, which are predominantly mediated by photoreceptors and bipolar cells, the candidate locus of the non-linearity is in the photoreceptoral (rod and cone) level or bipolar cell level.

Since we did not find consistent beat responses in the cross photoreceptor type condition, we cannot make further inferences about the presence of non-linear rod-cone interactions. In this work we examined beats for sinusoidal stimuli with same phase. A possible way to analyze rod-cone interactions is by systematically changing the phase difference between the rod and cone photoreceptor modulations, in conditions where beat responses are elicited. The presence of non-linear pupil responses in the outer retina may have applications in the study of retinal degenerations involving rods and/or cones, with different diseases (e.g., Retinitis pigmentosa, age-related macular degeneration) expected to have different signature beats depending on the degree of photoreceptor degeneration.

## Author Contributions

PB and DC conceived, designed and performed the experiments, and wrote the manuscript. JM and AZ critically revised the manuscript. All authors approved the final manuscript version.

### Conflict of Interest Statement

The authors declare that the research was conducted in the absence of any commercial or financial relationships that could be construed as a potential conflict of interest.
